# dAMN: a genome-scale neural-mechanistic hybrid model to predict bacterial growth dynamics

**DOI:** 10.1093/bioinformatics/btag230

**Published:** 2026-05-08

**Authors:** Jean-Loup Faulon, Danilo Dursoniah, Paul Ahavi, Antoine Raynal, Enrique Asin-Garcia

**Affiliations:** University Paris Saclay, INRAE, AgroParisTech, MICALIS Institute, Jouy-en-Josas, 78350, France; University Paris Saclay, INRAE, AgroParisTech, MICALIS Institute, Jouy-en-Josas, 78350, France; University Paris Saclay, INRAE, AgroParisTech, MICALIS Institute, Jouy-en-Josas, 78350, France; Bioprocess Engineering Group, Wageningen University & Research, Wageningen, 6700 AA, The Netherlands; Bioprocess Engineering Group, Wageningen University & Research, Wageningen, 6700 AA, The Netherlands

## Abstract

**Summary:**

This study presents dAMN, a genome-scale neural–mechanistic hybrid model that combines neural networks with dynamic flux balance analysis to predict bacterial growth dynamics across diverse nutrient environments. Using a residual network architecture, dAMN predicts reaction fluxes and lag-phase parameters from initial medium composition, then integrates these predictions under stoichiometric constraints derived from genome-scale metabolic models. Trained on *Escherichia coli* and *Pseudomonas putida* growth datasets across combinatorial media, dAMN accurately forecasts temporal growth dynamics and generalizes to unseen media conditions, with mean R² ≥ 0.9. The model also reproduces biologically relevant behaviors including substrate depletion, acetate overflow, and diauxic shifts, while explicitly modeling lag phases usually absent from standard dFBA.

**Availability and implementation:**

The dAMN software, associated models, and datasets are available at https://github.com/brsynth/dAMN-main-release and via Zenodo DOI: 10.5281/zenodo.17908125.

## 1 Introduction

A central challenge in modeling microbial metabolism is predicting growth dynamics across diverse nutrient environments. Most of the mechanistic and hybrid approaches struggle to generalize across media and rarely capture lag-phase adaptation. Dynamic Artificial Metabolic Networks (dAMN) address this gap. By embedding neural network components within a mechanistic dynamic flux balance analysis (dFBA) framework, dAMN learns substrate-dependent flux adjustments and lag-phase delays directly from nutrient environments while preserving the stoichiometric constraints of FBA. This enables accurate prediction of full growth curves including lag-phases across diverse media conditions.

Mathematical modeling of cellular metabolism has traditionally relied on kinetic models or constraint-based approaches. While kinetic models provide mechanistic accuracy, they require extensive parameterization and are not scalable to genome-scale networks. In contrast, Flux Balance Analysis (FBA) offers a scalable, stoichiometry-based framework for computing optimal steady-state flux distributions without requiring kinetic parameters (Orth et al. 2010). However, its steady-state assumption precludes direct simulation of batch or fed-batch processes, where extracellular conditions and intracellular fluxes change over time.

Dynamic Flux Balance Analysis (dFBA) extends FBA to temporal processes. Originally outlined by Varma and Palsson (1994) and formalized by Mahadevan et al. (2002) dFBA iteratively solves a sequence of FBA problems alongside integration of extracellular metabolite concentrations. Although dFBA captures time-varying behaviors, it often produces biologically unrealistic instantaneous shifts in metabolic fluxes and faces numerical stiffness.

To address these issues, several extensions have been proposed. Enzyme-constrained FBA (ecFBA) limits reaction rates based on enzyme capacity (Sánchez et al. 2017), while enzyme-change constrained models (Karlsen et al. 2023) explicitly bound the rate at which proteome fractions reallocate. Such models better reproduce phenomena like diauxic lags in *E. coli*, however these models require additional data beyond media composition. Other efforts extend dFBA to microbial consortia (Stolyar et al. 2007, Tzamali et al. 2011) or combine it with statistical surrogates (Negahban et al. 2025).

Recently, hybrid methods that integrate data-driven machine learning with mechanistic modeling have emerged as alternatives to purely kinetic or constraint-based approaches. Physics-Informed Neural Networks (PINNs) embed ODE systems directly in neural architectures, enabling estimation of hidden dynamics and parameters from sparse data (Raissi et al. 2019, Yazdani et al. 2020). PINNs require an explicit ODE model for growth that is rarely available at the genome scale. Neural-ODE based metabolic models (Rathod et al. 2024) are genome scale, they infer latent dynamic states from gene expression and decode them into fluxes but cannot reproduce growth curves. Artificial Metabolic Networks (AMNs), also genome scale, embed neural networks inside the FBA framework to improve flux prediction while reducing data requirements (Faure et al. 2023), however, AMNs do not handle metabolite or biomass concentrations. Other neural surrogates have been proposed, among these: Song et al. (2025) trains ANNs as FBA surrogates for Shewanella oneidensis in reactive-transport models, Dehkordi et al. (2023) uses convolutional networks to simulate Saccharomyces cerevisiae growth for fast control, COSMIC-dFBA (Gopalakrishnan et al. 2024) couples a data-driven cell-state classifier with dFBA to predict time-resolved metabolite profiles, and Negahban et al. (2025) constrain dFBA with partial least squares regression on kinetic parameters. However, these methods typically operate on fixed or narrow sets of substrates, treat the machine learning and dFBA components separately, or do not explicitly simulate extracellular metabolite depletion and growth trajectories, so they cannot systematically predict lag phases or generalize growth behavior across diverse media compositions.

To palliate these shortcomings, we present dynamic Artificial Metabolic Networks (dAMN), a hybrid model combining neural networks with mechanistic constraints from dFBA. Trained on experimental growth curves across combinatorial nutrient mixtures, dAMN predicts reaction fluxes and lag-phase dynamics from initial conditions and integrates these to produce full growth trajectories. We detail the model architecture, training procedure, and predictive performance in the following sections. Additional methods and results can be found in the Supplementary Information (SI), and links to the SI are provided whenever necessary.

## 2 Methods

The methodological approach presented here is structured into three distinct subsections. The first subsection describes the dAMN model, detailing its hybrid neural-mechanistic architecture, key equations, and loss functions used for training and predictions. The second subsection summarizes the experimental procedures used to generate the training datasets, including bacterial culture preparation and growth monitoring. The final subsection outlines the construction and the use of distinct training and test datasets for model validation.

### 2.1 dAMN model

The overall architecture of the dAMN model is illustrated in Fig. S1 in SI. At its core, dAMN is a hybrid neural-mechanistic model integrating neural networks with dFBA. The neural network components ($f_{\theta_{\mathrm{lag}}},\;f_{\theta_{\mathrm{lag}}}$) are designed to predict lag phase parameters ($t_{\mathrm{lag}},\;k_{\mathrm{lag}}$) and reaction fluxes (V(t)), from medium composition ($C_{\mathrm{true}}(0)$). The mechanistic component applies stoichiometric and kinetic constraints to these flux predictions, ensuring biological plausibility. The model can be formalized through the following equations:


(1) $$C(0)=C_{\mathrm{true}}(0)$$

(2) $$t_{\mathrm{lag}},\;k_{\mathrm{lag}}=f_{\theta_{\mathrm{lag}}}(C(0))$$

(3) $$V(t)=f_{\theta_{V}}(C(t-1))$$

(4) $$r(t)=1-\;e^{{-k}_{\mathrm{lag}}t}\;\mathrm{for}\;t\leq \;t_{\mathrm{lag}},\;1\;\mathrm{otherwise}$$

(5) C(t)=C(t-1)+r(t) S V(t) Δt

where:


$$t:\;\mathrm{discrete}\;\mathrm{time}\;\mathrm{in}\;[0,t_{\max}]\;\mathrm{with}\;\mathrm{constant}\;\mathrm{spacing}\;\mathrm{by}\;\Delta t$$

$$C_{\mathrm{true}}(t):\;\mathrm{measured}\;\mathrm{concentration}\;\mathrm{at}\;\mathrm{time}\;t\;\mathrm{for}\;\mathrm{all}\;\mathrm{reference}$$

metabolites (biomass included)

C(t): learned concentration at time t for all metabolites

V(t): learned flux at time t for all reactions

r(t): a exponential function r(t)=0 at t=0

$$\mathrm{and}\;r(t)=1\;\mathrm{for}\;t\geq t_{\mathrm{lag}}$$

$$t_{\mathrm{lag}}\text{:}\;\mathrm{learned}\;\mathrm{lag}\;\mathrm{time}$$

$$k_{\mathrm{lag}}:\;\mathrm{learned}\;\mathrm{stiffness}\;\mathrm{of}\;\mathrm{the}\;\mathrm{exponential}\;\mathrm{function}\;r(t)\;$$

$$f_{\theta_{\mathrm{lag}}}:\;\mathrm{feedforward}\;\mathrm{neural}\;\mathrm{network}\;\mathrm{with}\;\mathrm{parameters}\;\theta_{\mathrm{lag}}$$

$$f_{\theta_{V}}:\;\mathrm{feedforward}\;\mathrm{neural}\;\mathrm{network}\;\mathrm{with}\;\mathrm{parameters}\;\theta_{V}$$

S: stoichiometric matrix

Let us note that for internal metabolites C(t)=C(t-1) as S V(t)=0 consequently Equation (5) can be written as:


(6) $$C(t)=C(t-1)+r(t)T\;V(t)\;\Delta t\;\mathrm{for}\;t\;\mathrm{in}\;[1,\;t_{\max}]$$

where T, derived from the stoichiometric matrix S, can be viewed as a transport matrix mapping reactions to external metabolites, T=-1 (+1) for inward (outward) reactions.

The concentration update in Equation (6) follows a Euler integration scheme, where C(t-1) acts as the current state and r(t) T V(t) Δt as the incremental update. This formulation is analogous to a residual network (ResNet) design (He et al. 2016), where the current state plays the role of the skip connection and the flux network learns only the residual correction, facilitating gradient flow during training. The lag factor r(t) further acts as a time-dependent gate on this residual, analogous to gating mechanisms in recurrent architectures.

As in PINN we train our dAMN model in order to match both the observation (measured concentrations) and mechanistic constraints (dFBA constraints). The loss function given below reflect this multi-objective.


(7) $$\mathrm{Loss}=\sum_{t}\sum_{i}{w_{i}(t)\mathrm{Loss}}_{i}(t),\;t\in [1,\;t_{\max}],\;i=1,\ldots ,4$$

(8) $$\mathrm{Los}s_{1}(t)=\mathrm{mse}(C(t),C_{\mathrm{true}}(t))$$

(9) $$\mathrm{Los}s_{2}(t)=\mathrm{mse}(\mathrm{Relu}(C_{\mathrm{biomass}}(t)-C_{\mathrm{biomass}}(t-1)),0)$$

(10) $$\mathrm{Los}s_{3}(t)=\mathrm{mse}(\mathrm{SV}(t),0)$$

(11) $$\mathrm{Los}s_{4}(t)=\mathrm{mse}(\mathrm{Relu}(-V(t)),0)$$

The loss function enforces predicted concentrations match measured concentrations ($\mathrm{Los}s_{1}$), biomass concentrations cannot reduce over time ($\mathrm{Los}s_{2}$), the stoichiometric constraint is verified ($\mathrm{Los}s_{3}$), and all reactions are irreversible and therefore all fluxes are positive ($\mathrm{Los}s_{4}$). The weights, $w_{i}(t),\;$are specific to each loss term and evolve over time. Several functions, linear, exponential, cosines and sigmoid have been tested. Best results were obtained with the following exponential decay function:


(12) $$w_{i}(t)=\lambda_{i}e^{-k_{i}t},\;\;\;i=\;1\ldots 4$$

To determine the optimal values for the loss weighting ($\lambda_{i}$) and decay parameters ($k_{i}$) in the loss function, we performed hyperparameter optimization (see Results section).

We tested alternative architectures, in particular to model the lag-phase using a Hill function as in Flassig et al. (2016), and to remove time decay in the loss function. These are described in SI Section 1 (Figs. S1–S3). In general, these alternatives do not outperform the architecture described by Equations (1)–(12).

### 2.2 Experimental datasets

We acquired three experimental datasets. The first one (referred as M28) is composed of *E. coli* MG1655 growth curves acquired on 280 different culture media. The second (named putida) is composed of *P. putida* KT2440 growth curves acquired on 81 different culture media. In both cases the strains were grown on an M9 minimal medium supplemented with combinations of sugars (D-glucose, D-xylose, succinate), 20 amino acids, and 5 nucleobases. Further details on experimental procedures are provided in SI Section 2 for *E. coli* and SI Section 3 for *P. putida*. The third dataset (referred as Millard) was extracted from Millard et al. (2021). This dataset comprised concentration measurements at various timepoints for glucose, extracellular acetate and biomass for *E. coli* MG1655 grown on an M9 minimal medium supplemented with glucose and acetate. Additional information for that dataset is provided in SI Section 4.

### 2.3 Training and test sets

Two training and test sets were generated for both *E. coli* (M28) and *P. putida*. In the first set, the forecast set, dAMN was trained using two-thirds of the growth time points from all available data, and predictions were made on the remaining one-third. In the second set, the media set, dAMN was trained on two-thirds of the available media using all time points, and predictions were made for media conditions not included in the training set. In addition, two training/testing datasets were generated using the *E. coli* Millard data mentioned above. In the first case, the training set was composed of measured concentrations for extracellular acetate and biomass (with glucose concentrations in test set), and in the second case the training set was composed of measured concentrations for glucose and biomass (with acetate concentrations in test set). Additional information for these datasets are provided in SI Section 4.

## 3 Results

Prior to evaluating dAMN, we fine-tuned its architecture and hyperparameters. A critical dAMN parameter is the stoichiometric matrix used to build the mechanistic constraints. As we tested dAMN with *E. coli* MG1655 the stoichiometric matrix was obtained from *E. coli* genome-scale metabolic model iML1515 (Monk et al. 2017). This metabolic model comprises 1877 metabolites, 2712 reactions, and 1516 genes. To ensure all reactions were irreversible, reversible reactions were duplicated (one forward, one reverse) following the approach described in Faure et al. (2023), resulting in 3682 irreversible reactions and a stoichiometric matrix of dimensions [1877, 3682]. To construct the transport matrix *T*, we extracted rows and columns from the stoichiometric matrix corresponding to external metabolites, resulting in a transport matrix of dimensions [29, 3682]. The 29 matrix rows included the 28 medium metabolites and the biomass for which concentration was monitored. The “*reversible reaction duplicated*” iML1515 model was used for the *E. coli* M28 and Millard datasets mentioned earlier. For the Millard dataset extracellular acetate was added to the transport matrix. The same procedure was applied to *P. putida* model iJN1463 resulting in a stoichiometric matrix of dimensions [2153, 4034] and a transport matrix of dimensions [29, 4034].

**Figure 1:**
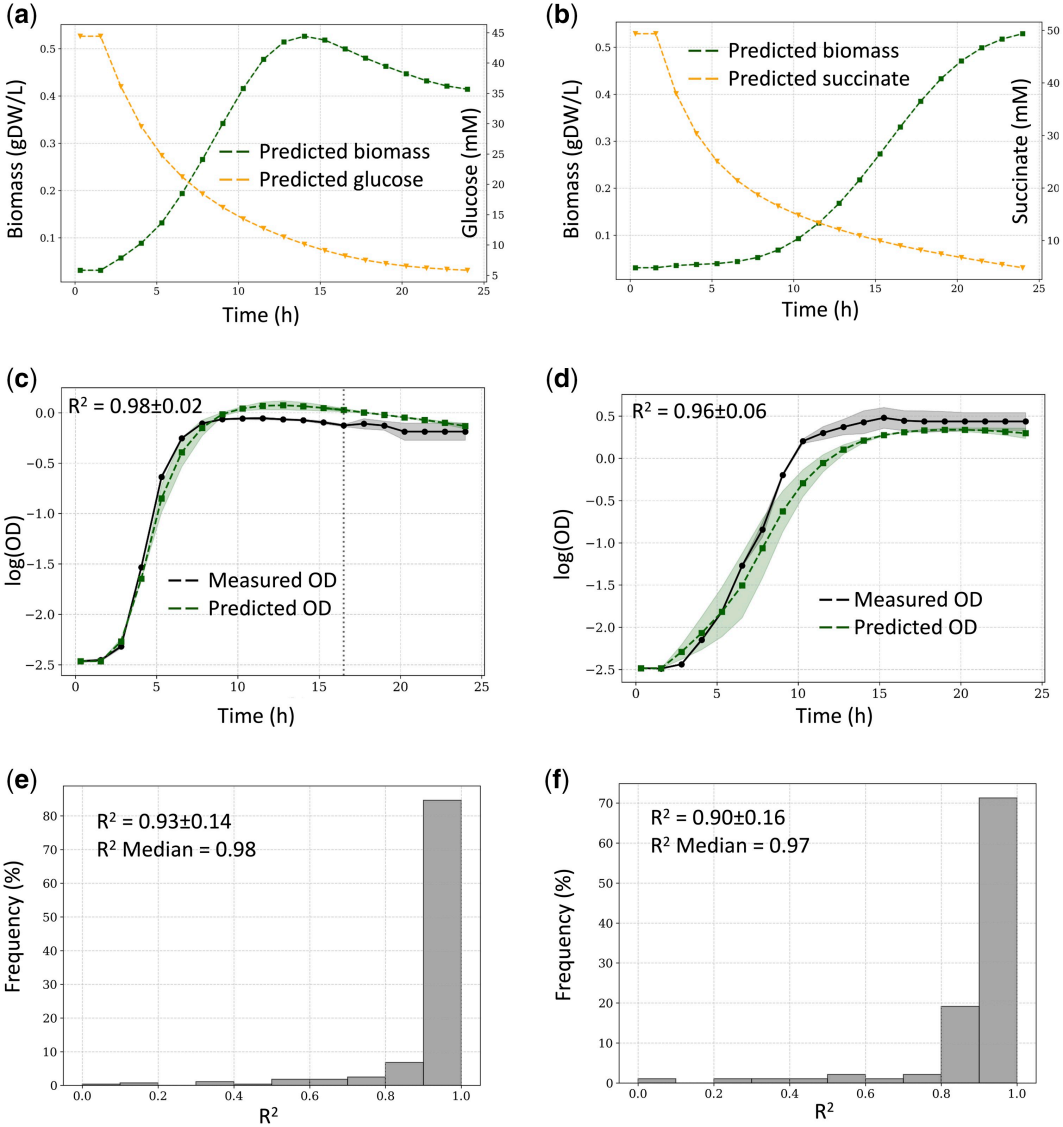
dAMN results for *E. coli* M28 dataset. (a) Predicted concentrations for biomass and D-glucose when the strain is grown on M9 with D-glucose only (medium number 1). (b) Predicted concentrations for biomass and succinate when the strain is grown on M9 with succinate only (medium number 156). (c) Example of a predicted growth curve compared to measured growth curve using the forecast training set (dashed line indicates when forecast begins). dAMN is trained on the first two-thirds of the datapoints, with predictions made for the final third. (d) Example of a predicted growth curve compared to measured growth curve using the media training set. dAMN is trained on the first two-thirds of all media, and predictions are made for media not used during training. Displayed curve represents prediction for a medium not present in the training set. (e) Distribution of R^2^ values between measured and predicted growth curves for the forecast training set. (f) Distribution of R^2^ values between measured and predicted growth curves for the media training set. Standard deviations (shaded areas in panels c and d) were obtained using three replicates for the experimental data, and running three models trained on the forecast and medium set using the top parameter set of Table S1.

We also searched the optimal loss weighting (*λ*_i_) and decay parameters (*k*_i_) of Equation (12), performing hyperparameter optimization. To that end, a coarse random search was conducted over log-uniform ranges for *λ*_i_ ([0.001, 0.01, 0.1, 1.0]) and linear for *k*_i_ ([0.0, 0.25, 0.5, 0.75, 1.0]), training each model for 1000 epochs with early stopping. Each candidate was evaluated using the medium set (3-fold cross validation), and the average regression coefficient (R^2^) between predicted and experimentally measured growth on the test set served as the metric for model selection. The 10 best-performing parameter sets achieving the highest average and median test R^2^ are summarized in SI Table S1. The top optimal parameter set was subsequently used for further analyses. All experiments were conducted using fixed random seeds and consistent data splits to ensure reproducibility.dAMN was first evaluated using the *E. coli* M28 and *P. putida* datasets mentioned earlier. In both cases, the forecast set and the media set were used to assess dAMN capacities for forecasting metabolite concentrations over time and predicting bacterial growth on unseen media conditions. Results are presented for *E. coli* M28 dataset in Fig. 1 and in SI Fig. S8 for *P. putida*. Figure 1a and b shows that the concentrations of substrates (glucose and succinate) follow expected biological behaviors, demonstrating gradual depletion corresponding to biomass accumulation. The forecast set results indicate strong predictive capability, exemplified by the close match between predicted and experimentally measured growth curves (panel c), with a median R^2^ of approximately 0.98 (panel e). The media set, which evaluates dAMN’s generalization to unseen media conditions, also exhibits robust predictions, as illustrated by the representative growth curve (panel d) and a median R^2^ value of around 0.97 (panel f).

As shown in SI Table S2, dAMN performance exhibits a moderate decline in predictive accuracy as the amount of training data was reduced (dropping from a mean R^2^ = 0.90 with 187 media in the training set to a mean R^2^ = 0.85 with only 93 media in training). Poorly performing examples (*cf*. Fig. S4 in SI) are mostly due to undetectable lag phases or sharp biomass drops during the stationary phase. Yet, dAMN appears to handle well different lag-phase lengths (Fig. S5 in SI).

Furthermore, as shown in Fig. S6 in SI, the lag times predicted by dAMN across unseen media reveal biologically meaningful substrate-dependent patterns. In particular, media containing amino acids as the primary nutrient exhibit substantially shorter lag phases (∼1.5 h) than media containing sugars (∼5.6 h). This is consistent with reports that amino-acid-rich conditions in *E. coli* support rapid growth with preferred amino-acid catabolism, reduced glucose uptake, and altered overflow metabolism (Zampieri et al. 2019), whereas adaptation to amino-acid-poor media requires broader biosynthetic proteome reallocation (Wu et al. 2023).

Beyond growth curves, dAMN can also predict growth rates across unseen media, with predicted and measured values in good agreement (R^2^ = 0.86, Fig. S7 in SI), consistent with growth rate predictions previously reported for AMNs (Faure et al. 2023). These results collectively validate the model’s predictive accuracy and its generalization capacity to various nutritional contexts.

Results similar to those presented in Fig. 1, were obtained with the *P. putida* dataset for both forecasting (*cf.*  Fig. S8c in SI where mean R^2^ = 0.94, median R^2^ = 0.98) and predicting growth curves of unseen media (*cf*. Fig. S8d in SI where mean R^2^ = 0.90, median R^2^ = 0.94).

Because dAMN is able to predict substrate consumption concurrent with biomass increase (*cf*. Fig. 1a and b), we sought to assess this emergent behavior quantitatively. To this end, we used the Millard dataset and trained dAMN on biomass and extracellular acetate concentrations. Fig. S9a in SI shows that glucose concentrations are accurately reconstructed under these conditions. Likewise, dAMN accurately predicts acetate overflow when trained on glucose and biomass concentrations (Fig. S9b in SI). We compared these results (Section 4 in SI) with those obtained with a PINN based on the ODE model of Millard (Millard et al. 2021). As is customary with PINNs, our PINN was trained both to fit the measured concentrations and to infer values for unknown kinetic parameters (Table S3 in SI). Fig. S9c and d, show that dAMN outperforms PINN unless precise and accurate values for the unknown kinetic parameters are provided (Fig. S9e and f).

To further investigate substrate consumption hierarchies, we trained dAMN exclusively on biomass and glucose time series, without providing acetate measurements during training, and extended the predictions beyond the experimental time window. In these forward simulations, dAMN predicted acetate consumption following glucose depletion, accompanied by continued biomass increase, consistent with classical diauxic behavior (Fig. S10 in SI), even though these phases were not present in the training data. These results indicate that dAMN can infer and reproduce multi-substrate utilization patterns from limited supervision.

## 4 Discussion and conclusion

This study introduces dAMN, a hybrid neural–mechanistic model that integrates data-driven neural networks with mechanistic constraints from genome-scale metabolic networks to predict the full dynamics of microbial growth curves. The model was evaluated using two distinct test sets: a forecast set to assess temporal extrapolation, and a media set to evaluate its capacity to generalize to entirely unseen nutritional compositions. In both cases, dAMN demonstrated high predictive accuracy, achieving average R^2^ values ≥ 0.90 for both forecast and media sets acquired for *E. coli* (Fig. 1) and *P. putida* (Fig. S8 in SI). These performances confirm that the model not only learns realistic time dynamics but also generalizes robustly across diverse and previously unobserved environmental conditions.

As with AMNs relative to FBA (Faure et al. 2023) a key advantage of dAMN over standard dFBA is its ability to infer uptake fluxes directly from extracellular medium concentrations, rather than requiring them to be measured or specified a priori. This data-driven estimation of uptake improves the accuracy of growth predictions, as illustrated by the dAMN–dFBA comparison carried out in Section 5 and Fig. S11 in the SI.

A particularly noteworthy outcome of our results is the predicted depletion pattern of substrates like glucose and succinate, as shown in Fig. 1a and b. These results were obtained despite the fact that only the initial substrate concentrations were provided and no intermediate data was used during training. The model was nevertheless able to produce realistic substrate consumption curves that mirror biomass accumulation. This emergent behavior arises solely from the enforcement of the stoichiometric constraint SV(t)=0, which ensures internal metabolite balance. Since substrate consumption is the only mechanism available to fuel biomass production, the model was constrained to infer realistic usage rates, an interesting outcome given the lack of direct supervision on substrate dynamics. This observation was quantitatively validated with the *E. coli* Millard dataset where glucose consumption and acetate overflow could accurately be predicted when compared to measurements (Section 4 and Fig. S9 in SI). Additionally, with forward simulations, dAMN was able to predict acetate consumption following glucose depletion (Fig. S10 in SI), even though this diauxic shift was absent from the training data.

The explicit modeling of the lag phase via exponential or Hill functions is another feature distinguishing dAMN. Classical dFBA implementations typically neglect this initial adaptation delay (*cf*. Fig. S11 in SI). While multiphase models based on empirical phase divisions (Moimenta et al. 2025) or enzyme-change constraints Sánchez et al. (2017) and Karlsen et al. (2023) have addressed lag phases, they often lack fully data-driven parametrization. Approaches incorporating kinetic delays or dynamic objectives (Waldherr et al. 2015) similarly offer conceptual parallels but typically require more complex mechanistic assumptions. Our approach aligns with these multiphase modeling strategies but is notably simpler, entirely data-driven, and implicitly captures biological adaptation without explicitly modeling regulatory or proteomic shifts.

Furthermore, dAMN uniquely predicts complete growth curves across unseen media, overcoming limitations of previous hybrid models. All the hybrid approaches mentioned in the introduction either lack generalization to new nutrient contexts, omit full dynamic modeling of biomass and metabolites, or rely on rich omics datasets not always available in experimental workflows. dAMN stands out in that it uses only initial metabolite concentrations, applies physics-inspired constraints, and can accurately extrapolate to forecast trajectories and to combinatorial media not seen during training.

## Supplementary Material

btag230_Supplementary_Data
